# Impacts of kinetin implementation on leaves, floral and root-related traits during seed production in hybrid rice under water deficiency

**DOI:** 10.1186/s12870-023-04405-6

**Published:** 2023-08-22

**Authors:** Mohamed I. Ghazy, Hassan Sh. Hamad, Elsayed E. Gewaily, Eman M. Bleih, Elsayed F. A. Arafat, Wael H. EL-Kallawy, Sabry A. EL-Naem, Medhat Rehan, Khairiah Mubarak Alwutayd, Diaa Abd El Moneim

**Affiliations:** 1 Rice Research and Training Department, Field Crops Research Institute, Agricultural Research Center, Kafrelsheikh, 33717 Egypt; 2 Department of Plant Production and Protection, College of Agriculture and Veterinary Medicine, Qassim University, 51452 Buraydah, Saudi Arabia; 3 Department of Genetics, Faculty of Agriculture, Kafrelsheikh University, Kafr El-Sheikh, 33516 Egypt; 4 Department of Biology, College of Science, Princess Nourah bint Abdulrahman University, P.O. Box 84428, 11671 Riyadh, Saudi Arabia; 5 Department of Plant Production (Genetic Branch), Faculty of Environmental Agricultural Sciences, Arish University, El-Arish, 45511 Egypt

**Keywords:** CMS lines, Growth traits, Drought stress, Kinetin, Plant root, Sustainability

## Abstract

**Background:**

Water deficit is one of the most significant abiotic factors affecting rice and agricultural production worldwide. In hybrid rice, cytoplasmic male sterility (CMS) is an important technique for creating high-yielding crop based on heterosis. The phytohormone kinetin (Kin) regulates cell division in plant during the early stages of grain formation, as well as flow assimilation and osmotic regulation under water stress. The present study performed to estimate the effects of irrigation intervals (irrigation each six days (I_6_), nine days (I_9_), twelve days (I_12_) and fifteen days (I_15_) against continuous flooding (CF, each three days)) and kinetin exogenously application (control, 15 mg L^−1^ and 30 mg L^−1^) on hybrid rice (L1, IR69625A; L2, G46A and R, Giza 178 R) seed production.

**Results:**

Leaves traits (Chlorophyll content (CHC), relative water content (RWC), stomatal conductance (SC), Leaf temperature (LT) and transpiration rate (TR)), floral traits such as style length (SL) and total stigma length (TSL), in addition to root traits (*i.e.,* root length (RL), root volume (RV), root: shoot ratio (RSR), root thickness (RT), root xylem vessels number (RXVN) and root xylem vessel area (RXVA) were evaluated and a significant enhancement in most traits was observed. Applying 30 mg L^−1^ kinetin significantly and positively enhanced all growth, floral and roots traits (RV and RXVA recorded the most increased values by 14.8% and 23.9%, respectively) under prolonging irrigation intervals, in comparison to non-treated plants.

**Conclusions:**

Subsequently, spraying kinetin exogenously on foliar could be an alternative method to reduce the harmful influences of water deficiency during seed production in hybrid rice.

**Supplementary Information:**

The online version contains supplementary material available at 10.1186/s12870-023-04405-6.

## Introduction

Rice is highly sensitive to water shortage and climate change particularly in response to extended water deficit periods and temperature increases. Rice (Oryza sativa L.) considers one of the most important cereal crop for humans, providing 20% of the world's calorie needs and 13% of its protein requirements [1]. Rice not only provides the majority of energy, but it also contains protein, vitamins, and other nutrients [2]. It is a semi-aquatic crop plant that needs a lot of water to grow and develop properly. During the summer, it takes up to 22% of Egypt's planted area [3]. Hybrid rice produces 20–30% more than traditional rice varieties which considers a priority in food security for many people around the world. There are several constraints that limit the hybrid rice production, *i.e.,* lack of acceptability in some regions, higher seed production cost, limitation of participating the public sector corporations in seed production and lack of heterosis with higher level. Efficient of seed production is a major requirement and determine the success and sustainability of hybrid rice production. Furthermore, Egypt is regarded as one of the first countries to introduce hybrid rice technology [4, 5].

Water deficit (drought) has an injurious effect on crop production, environment and economy. Drought combining with climate change has affected many regions around the world that will affect negatively on crop production and global food security [6]. These obstacles decrease the cultivation area and subsequently the future global production [7]. Water scarcity affects plant development and productivity in general by causing morphological, biochemical, and physiological alterations in crops. Plants that are significantly stressed by drought eventually die because photosynthesis and metabolism are substantially interrupted [8–11]. Furthermore, water shortage inhibits cell enlargement and plant growth that will influence physiological processes, including photosynthesis, respiration, ion uptake, carbohydrate and nutrient metabolism [8, 9]. Water deficit decreases water uptake and seedling vigour throughout the germination stage. At the cell level, drought disrupts the metabolic process, reduces ATP synthesis, and inhibits water balance, resulting in poor seed germination [12–14].

The water limitation period and its severity determine the extent of loss in yield through reducing the life cycle and grain filling period. In rice, grain weight, panicle number, and yield considered the most effective parameters that affected grain yield. Besides, yield components and grain yield have inspected in relation to water deficit occurring at various stages of growth in rice. The crop production and plant biomass and related traits (such as biomass at harvest, number of panicles per unit area (PNPU), panicle length (PL), number of paddies per panicle (GNPP), 1000-grain weight (GW), and filled grain percentage (FP) reduced due to alteration in growth characteristics caused by water shortage [12, 13, 15–17].

Stigma exertion, one of the stigma morphological indices that plays a significant role in determining the rice mating system, is widely implemented in seed production from hybrid rice based on its pollination advantages. However, it is still unclear how stigmatization affects rice's pollination and spikelet fertility when there is a lack of water [18].

Plant growth hormones are small natural molecules that stimulate many aspects during plant growth and differentiation. Phytohormones are important in mediating different responses during stress cues for growth and developmental control during biotic and abiotic stresses [19, 20]. Up to date, nine classes of phytohormones have been determined such as cytokinins (CK), auxins, abscisic acid, (ABA), gibberellins (GA), salicylates (SA), ethylene (ET), jasmonates (JA), strigolactones (SL) and brassinosteroids (BR) [21]. Cytokinins (CKs) stimulate the cell division that influence the growth of plant. They are synthesis in plant roots from the adenine precursor. Additionally, they move in the woody tissue (xylem) towards leaves and fruits (the place of their use for plant growth and differentiation of cells). Furthermore, plant hormone interactions may play a key impact in regulating cellular processes such as cell elongation and spikelet degeneration [22, 23].

Production of hybrid rice seeds necessitates a high level of technological expertise. A sufficient amount of irrigation and other agronomic techniques are necessary to fully utilise the potential of hybrid rice cultivars [24–27]. Various traits of plant development, physiology and growth alter under cytokinin application (*i.e.,* flower and fruit development, plant-pathogen-interactions, seed germination, leaf senescence, and apical dominance). CKs have a vital role in controlling the development and growth of plants, as well as improving plant tolerance to water deficit. They improve photosynthesis by stimulating cell division and increasing sink strength. CK is inactivated and metabolized via degradation by CK oxidase enzymes or by conjugation with carbohydrates (sugars) [20, 22–25]. Kinetin (Kn, a type of cytokinines) enhances chlorophyll content in plants by stimulating the production of photosynthetic proteins, accelerates the division in cells, and modifies the apical dominance in plants [28, 29].

Our aims in the present study were: (1) to applicate an external kinetin phytohormone at different water deficit conditions, (2) to evaluate the possible effects of exogenous kinetin implementation on leaves, roots, floral and yield-related traits, (3) assessing the association between applied treatments and the evaluated traits, (4) exploring the best CMS lines that may incorporate in seed production of hybrid rice under drought stress.

## Materials and methods

### CMS lines and experiment conditions

Two cytoplasmic male sterility lines (CMS lines) in addition to one restore line were selected for the present study. L1 (IR69625A, recorded genotype code A1, days to heading recorded 104.5, Wild abortive (WA) CMS line cytoplasmic source and originated from IRRI), whereas L2 (G46A, coded A2, days to heading reached 88.9, Gambiaca CMS line cytoplasmic source originated from China). In the experimental farm of Rice Research and Training Center (RRTC, 31°08'N and 30°58'E)-Sakha-Kafr El-Sheikh-Egypt, the two CMS lines planted in two seasons (2020 and 2021) under exogenously implemented of kinetin combined with water deficit intervals. The used restore line (Giza 178R, coded with R, days to heading measured 100.7, cytoplasmic source (Giza175/ Milyang 49 Indica/Japonic type) and origin from Egypt.

The implemented irrigation intervals were as follow: CF = continuous flooding, I_6_ = irrigation every six days, I_9_ = irrigation every nine days, I_12_ = irrigation every twelve days and I_15_ = irrigation every fifteen days. Besides, the exogenous kinetin treatment (control, 15 mg L^−1^, and 30 mg L^−1^) coupled with water shortage intervals were examined in related to growth, floral and root -related traits plus its reversal on F1 hybrid rice seed productivity.

### The implemented treatments and agricultural practices

In the current experiment, a strip-split plot design was used in three replications. In the horizontal plots, the irrigation treatments (CF, irrigation every 6, 9, 12, and 15 days) were placed. While the two CMS lines were separated the vertical plots (A1 and A2). Meanwhile, the three kinetin treatments (control, 15 mg L^−1^, and 30 mg L^−1^) were plotted in the split vertical plots. Deep channels encircled the horizontal plots to manage and prevent any lateral movement of irrigation.

The CMS lines seeds were soaked for 24 h in fresh water, then drained and incubated at a rate of 5 kg from the restorer line (Giza 178 R) and 15 kg ha^−1^ (15 kg from the CMS lines (IR69625A and G46A) for 48 h. CMS lines IR69625A (A1) and G46A (A2) were seeded on May 1st and 20th, respectively. To achieve optimum flowering synchronization, the restorer line (R) planted in three times. The first planting date was when 2.5 leaves of CMS line A1 recorded, the second date when leaves counted 3.5 in line A1, whereas the third time applied when number if leaves estimated 5 in the same CMS line. In the permanent field, fertilizing rice plants with nitrogen and phosphate performed at the rate of 165 kg N ha^−1^ and 240 kg ha^−1^ from urea (46% N) and single super phosphate (15.5% P_2_O_5_), respectively. A 50 kg ha^−1^ of 22% of ZnSO_4_ was supplied before planting and after pudding. All practices related to agronomy were performed as recommended whereas the average of climate temperatures and soil conditions are presented in Tables S1 and S2.

After thirty-day from sowing, both R and A line seedlings were transplanted in range 3–4 and 2 seedlings per hill, respectively. The distance between seedlings in row for A-A, R-R, and R-A lines was kept at 15, 20, 30 cm, respectively, while the hill distance among the R and A lines adjusted at 15 cm. Furthermore, CMS seed production demand a 100-m isolation area and R lines (20 rows) were planted around the experiment location to prevent cross pollination. Each major plot was separated by a plastic barrier (2.5 m high) to prevent transfer of pollen grain among treatments. Regular gibberellic acid (GA_3_) application in two times were applied: (1) GA_3_ with 40% sprayed on A lines at heading percentages 15–20, (2) GA_3_ with 60% performed at 35–40% heading of A lines (five days after heading). The shaking process achieved two-to-three times with bamboo sticks from the pollen parents (R line) for a period of 10 days to supply enough pollen grains.

### External application of kinetin

Two concentrations of Kinetin (15 and 30 mg per liter) were implemented twice as a foliar spray on the CMS lines during the mid tillering and panicle initiation stages in comparison to non-treated plants (control plants were treated with distilled water). Moreover, the irrigation periods were applied after transplanting with 15 days.

### The evaluated plant traits

#### Leaves growth, root, floral and seed yield parameters

Values were measured for leaves traits such as chlorophyll content (CHC, mg ds-1), relative water content percentage (RWC, %), stomatal conductance (SC, mmol m-2), leaf temperature (LT, ℃), and transpiration rate (TR, µg cm-2 s-1), style length (SL), total stigma length (TSL). Chlorophyll content (CHLC; SPAD unit) was recorded from the topmost completely expanded leaves on the main panicle during the flowering period, utilizing a SPAD meter (Model: SPAD-502, Hangzhou Mindfull Technology Co., Ltd., Hangzhou, China). Relative water content (RWC %) was calculated as previously stated by Barrs and Weatherley [30], from the flag leaf. For assessing the steady-state CO_2_ and H_2_O exchange degrees of leaves, the portable steady-state porometer LICOR (LI-1600, Lincoln, NE, USA) was used. Transpiration rate (TR µg cm^−2^ s^−1^) and stomatal conductance (SC mmol m^−2^) were assessed in the fully expanded flag leaf. The leaf temperature (LT, °C) was calculated from the steady-state porometer pressed against the adaxial and abaxial surfaces of the leaf by the thermocouple, whereas the leaf-to-air temperature gradient (TL–TA) was evaluated through the atmospheric temperature [31]. Floral traits observations were recorded from ten randomly selected spikelets for each genotype. Spikelets samples were immediately fixed in acetic-alcohol (actic acid:ethanol, 1:3) and kept in the fridge at four degrees until investigation [32]. Photographs of style length (mm) and pistil length (total stigma length, mm) were measured under ocular microscope at 10 × magnification to eyepiece micrometer and images were taken with DP70 digital camera attached to an Axioplane 2 microscope (Carl Zeiss, Germany) at 350 for floral traits.

Root characteristics of CMS lines including root volume (RV, cm^3^), root length (RL, cm), root: shoot ratio (RSR), root thickness (RT, mm), root xylem vessel area (RXVA, mm^2^) and root xylem vessels number (RXVN) were also evaluated under water scarcity and kinetin application. Root traits were measured using five plants/genotype at 24 days after stress imposition as described by Pantuwan et al. [33] and Gaballah et al., [34]. Root samples of 2 cm tall in average were taken from the nodal root tip for each root. Samples were immediately subjected for fixation and storage in FAA (formalin, 10%; acetic acid, 5%; ethyl alcohol, 50% and distilled water, 35%). Then, samples dehydrated with 50, 70 and 95% ethanol, followed by sectioning by the microtome (Reichert-Jung, Model 1130/Biocut) with 10 mm slice thickness at 20 mm distance from the root tip. After staining with safranine and fast green, photographs of root cross sections were taken by a microscope (Olympus BX51) since one pixel represented 0.47 mm. The average of root xylem vessels number (RXVN) was counted under the light microscope. The average of root xylem vessel area (RXVA, mm2) was measured under ocular microscope at 10 × magnification. The average diameter of all xylem vessels of the three roots/plant were transformed to area by using the formula:

$$Area= \pi \times r2$$

Where, $$\pi =Pi \left(3.14\right), r=radius$$.

Additionally, yield-related parameters such as seed yield (SY, t ha^−1^), seed set (SS, %) and harvest index (HI, %) were recorded under the implemented conditions. At 80% golden yellow color in seeds, the crop was harvested and seeds sun-dried up to 14% moisture to evaluate seed yield. Seed set percentage was calculated according to the following formula:

$$\mathrm{Seed}\;\mathrm{set}\;\%\;=\;\frac{\mathrm{Number}\;\mathrm{of}\;\mathrm{filled}\;\mathrm{grains}/\mathrm{panicle}}{\mathrm{Total}\;\mathrm{Spikele}t\;\mathrm{number}/\mathrm{panicle}}\;\times\;100$$

All the studied traits were estimated based on the Standard Evaluation System of IRRI (2014).

### Statistics analysis

The experiment was designed according to strip-split plot design in three replications. The obtained data analyzed based on the ANOVA technique using a statistical computer package COSTAT and the mean differences were compared by the Duncan’s Multiple Range Test [35]. Principal component analysis (PCA) and heatmap were conducted by the XLSTAT software version 2019 [36].

## Results

### Effects of drought stress, kinetin application and their interaction on leaves traits

The performance of CMS lines, IR69625A (L1) and G46A (L2) under irrigation intervals and kinetin employment as well as their interactions on the leaves traits including chlorophyll content (CHC), relative water content percentage (RWC%), stomatal conductance (SC), Leaf temperature (LT) and transpiration rate (TR), are presented in Table 1. Prolong the irrigation intervals exhibited highly negative and significant effects for all traits. Continuous flooding (CF) recorded the highest values for all evaluated traits whereas, the lowest values were assigned to I_15_ treatment except leaf temperature (LT) which showed opposite trend in both seasons.

**Table 1:** Effect of irrigation intervals, two CMS lines and kinetin application as well as their interactions on plant leave traits during 2020 and 2021 seasons

Main effect and interaction	Chlorophyll Content (mg ds^−1^)		Relative water content (% R.W.C)		Stomatal Conductance (mmol m^−2^ s^−1^)		Leaf Temperature (℃)		Transpiration Rate (µg cm^−2^ s^−1^)	
	2020	2021	2020	2021	2020	2021	2020	2021	2020	2021
Irrigation intervals (I)										
CF	42.40a	44.34a	68.09a	70.99a	0.085a	0.095a	27.14c	29.14b	45.63a	47.02a
I_6_	41.13b	42.22b	65.36b	67.25b	0.083b	0.093b	26.05d	28.05c	43.34b	44.73b
I_9_	39.01c	40.31c	63.32c	65.21c	0.078c	0.088c	25.23e	27.24d	42.74c	44.12c
I_12_	37.11d	37.91d	57.55d	59.44d	0.067d	0.077d	29.12b	31.12a	40.85d	42.23d
I_15_	36.41e	36.93e	56.64e	58.53e	0.060e	0.070e	29.82a	31.12a	40.15e	41.54e
F-test	**	**	**	**	**	**	**	**	**	**
Genotypes (G)										
L1	39.47b	40.59b	50.64c	52.73c	0.077b	0.087b	27.54b	29.54b	40.63b	42.01b
L2	40.39a	41.53a	51.48b	53.57b	0.082a	0.092a	28.38a	30.38a	38.05c	39.44c
Giza178R	37.77c	38.91c	84.45a	86.55a	0.065c	0.076c	26.48c	28.48c	48.94a	50.33a
F-test	**	**	**	**	**	**	**	**	**	**
Kinetin application (K)										
Control	38.30b	39.43c	61.20c	63.29c	0.072b	0.083c	27.11c	29.11c	42.14c	43.53c
15 mg L^−1^	39.38ab	40.52b	62.30b	64.39b	0.082a	0.085b	27.45b	29.45b	42.54b	43.93b
30 mg L^−1^	39.94a	41.08a	63.08a	65.17a	0.065c	0.086a	27.84a	29.84a	42.93a	44.32a
F-test	*	*	**	**	**	**	**	**	**	**
Interactions										
I × G	**	**	*	**	**	**	NS	NS	**	**
G × K	**	**	NS	NS	NS	NS	NS	NS	NS	NS
I × K	NS	NS	*	NS	NS	NS	NS	NS	NS	NS
I × G × K	NS	NS	NS	NS	NS	NS	NS	NS	NS	NS

^*^ = Significant at 0.05 level, ** = Significant at 0.01 level and *NS* Not significant. Means in the same column designated by the same letter are not significantly different at 5% level

Furthermore, the assessed L2 displayed the highest values for CHC, SC and LT with average 40.96 mg* ds*^*−1*^, 0.087 mmol* m*^*−2*^, and 29.38c, respectively, in the two assessed seasons, meanwhile, the increment in RWC% and TR were assigned to Giza 178 (R line) with average 85.50 mmol m^−2^ s^−1^ and 49.64 µg cm^−2^ s^−1^, respectively. Kinetin application at 30 mg L^−1^ had significant or highly significant and positive impacts on all evaluated traits in comparison with untreated plants. The average values of leaves traits in the two seasons of study were 40.51 mg ds^−1^, 64.13%, 0.083 mmol m^−2^ s^−1^ (under 15 mg L^−1^), 28.84 ℃ and 43.62 µg cm^−2^ s^−1^ for CHC, RWC, SC, LT and TR respectively. In contrast, untreated plants produced the lowest values of leaves traits reached 38.86 mg ds^−1^, 62,24%, 0.077 mmol m^−2^ s^−1^, 28.11 ℃ and 42.83 µg cm^−2^ s^−1^, respectively..

The interaction between the periods of irrigation and genotypes (I × G) showed a highly significant effect for all evaluated characteristics except LT. Going forward, interaction between the genotypes and kinetin spray (G × K) manifested highly positive and significant increment in CHC, whereas the other studied traits exhibited no significant change. On the other hand, the studied leaves traits were unaffected by the interaction between irrigation intervals and kinetin application (K x I) or among irrigation intervals, genotypes, and kinetin (I x L x K) treatments.

The analysis of results implied significant and highly significant impacts of interaction between irrigation periods and genotypes (I × G) on CHC, RWC, SC and TR under both seasons (Table 2). The best values of CHC were recorded by the three genotypes (L1, L2 and R) under continuous flooding (CF) without significant difference among them in the two seasons of comarison. Besides, L2 exhibited the greatest SC value (0.10 mmol m^−2^ s^−1^) in average under CF, whereas, Giza178R displayed the highest and significant valuse for RWC and TR under CF in both seasons. Otherwise, water deficit stress caused shortage in all growth traits compared to normal irrigation as clearly stated by irrigation every 15 day (I_15_) treatment in the three genotypes.

**Table 2:** Effect of interaction between irrigation intervals and genotypes on chlorophyll content, relative water content, stomatal conductance and transpiration rate during 2020 and 2021 seasons

Genotypes	Irrigation intervals	Chlorophyll content (mg/ds-1)		Relative water content (%)		Stomatal conductance (mmol m^−2^ s^−1^)		Transpiration rate (µg cm^−2^ s^−1^)	
						Years			
		2020	2021	2020	2021	2020	2021	2020	2021
L1	CF	42.50a	44.45a	56.84 g	60.50f	0.085d	0.095d	43.97f	45.36f
	I_6_	41.22c	42.32b	54.57 h	56.46 h	0.082e	0.092e	41.78 g	43.17 g
	I_9_	39.02de	40.40c	52.86i	54.75i	0.078f	0.088f	41.19 h	42.58 h
	I_12_	37.61f	38.42e	44.96 k	46.85 k	0.074 g	0.084 g	38.46j	39.85j
	I_15_	36.88f	37.40f	43.96 l	45.85 l	0.067i	0.076i	37.73 k	39.12 k
L2	CF	42.63a	44.58a	57.60f	60.50f	0.095a	0.105a	40.55i	41.94i
	I_6_	41.35bc	42.45b	55.29 h	57.18 h	0.092b	0.102b	38.53j	39.91j
	I_9_	39.22de	40.52c	53.57i	55.46i	0.087c	0.097c	37.98 k	39.37 k
	I_12_	39.75d	40.55c	45.81j	47.70j	0.070 h	0.080 h	36.95 l	38.34 l
	I_15_	39.02de	39.54d	45.15jk	47.03jk	0.063j	0.073j	36.22 m	37.61 m
Giza178R	CF	42.06ab	44.01a	89.83a	92.72a	0.076 g	0.086 fg	52.35a	53.74a
	I_6_	40.80c	41.90b	86.23b	88.12b	0.074 g	0.084 g	49.73b	51.12b
	I_9_	38.70f	39.99 cd	83.53c	85.42c	0.070 h	0.080 h	49.03c	50.42c
	I_12_	33.96 g	34.76 g	81.88d	83.77d	0.057 k	0.067 k	47.12d	48.51d
	I_15_	33.34 g	33.85 h	80.80e	82.69e	0.051 l	0.061 l	46.49e	47.88e

Means in the same column designated by the same letter are not significantly different at 5% level

The interaction between genotypes and kinetin treatment (G x K) is depicted in Table 3. When kinetin was applied at 15 mg L^−1^, Giza178R displayed a considerable increase in chlorophyll content with an average 41.83 mg ds^−1^. On the other hand, L1 sprayed with 30 mg kinetin L^−1^ exhibited the lowest CHC in both seasons.

**Table 3:** Effect of interaction between genotypes and kinetin application on chlorophyll content during 2020 and 2021 seasons

Genotypes	Kinetin application	Chlorophyll content (mg/ds^−1^)	
		Years	
		2020	2021
L1	Control	38.22d	39.36d
	15 mg L^−1^	39.31c	40.45c
	30 mg L^−1^	37.35e	38.49e
L2	Control	39.71c	40.38c
	15 mg L^−1^	40.61b	41.74b
	30 mg L^−1^	37.83de	38.96de
Giza178R	Control	40.75b	41.59b
	15 mg L^−1^	41.26a	42.39a
	30 mg L^−1^	38.12d	39.26d

Means in the same column designated by the same letter are not significantly different at 5% level

### Root traits in CMS lines under irrigation intervals, kinetin and their interaction

Root characteristics including, root length (RL, cm), root volume (RV, cm^3^), root: shoot ratio (RSR), root thickness (RT, mm), root xylem vessels number (RXVN) and root xylem vessel area (RXVA, mm^2^) were estimated under the effect of irrigation intervals, genotypes, and kinetin application as well as their interactions (Table 4). Continue exposure the genotypes under study to water deficit, significantly reduced the root traits (RL, RV, RSR, RT, RXVN and RXVA) by 27.2%, 37.2%, 12.7%, 30.5%, 10.8% and 32.2%, respectively.

**Table 4:** Effect of irrigation intervals, genotypes and kinetin application as well as their interactions on root traits during 2020 and 2021 seasons

Main effect and interaction	R L (cm)		R V (cm^3^)		R:S Ratio		RT		RXVN		RXVA (mm)	
							Years					
	2020	2021	2020	2021	2020	2021	2020	2021	2020	2021	2020	2021
Irrigation intervals (I)												
CF	25.37a	26.34a	49.02a	52.52a	0.51a	0.59a	0.93a	0.97a	5.33a	5.38a	0.57a	0.61a
I_6_	23.18b	24.16b	42.88b	46.38b	0.50b	0.58b	0.83b	0.87b	5.29a	5.34a	0.54b	0.58b
I_9_	21.23c	22.20c	38.08c	41.58c	0.49c	0.57c	0.73c	0.77c	5.22a	5.27a	0.47c	0.51c
I_12_	19.62d	26.35a	33.91d	37.41d	0.48d	0.56d	0.69d	0.73d	5.20a	5.22a	0.43d	0.47d
I_15_	18.34e	19.33e	30.14e	33.64e	0.44e	0.52e	0.64e	0.68e	4.76b	4.79b	0.38e	0.42e
F-test	**	**	**	**	**	**	**	**	**	**	**	**
Genotypes (G)												
L1	19.25c	20.23c	34.71c	38.21c	0.38c	0.46c	0.73c	0.76c	4.51c	4.56c	0.49a	0.53a
L2	22.34b	23.30b	39.36b	42.86b	0.48b	0.56b	0.83a	0.86a	5.51a	5.56a	0.48b	0.52b
Giza178R	23.05a	24.01a	42.35a	45.85a	0.58a	0.66a	0.75b	0.78b	5.47b	5.52b	0.46c	0.50c
F-test	**	**	**	**	**	**	**	**	**	**	**	**
Kinetin application (K)												
Control	20.25c	21.24c	35.80c	39.30c	0.46c	0.54c	0.73c	0.77c	4.96b	5.01b	0.41c	0.45c
15 mg L^−1^	21.70b	22.68b	38.32b	41.82b	0.48b	0.56b	0.76b	0.80b	5.23a	5.28a	0.48b	0.53b
30 mg L^−1^	22.68a	23.66a	42.31a	45.81a	0.50a	0.58a	0.81a	0.85a	5.30a	5.35a	0.55a	0.58a
F-test	**	**	**	**	**	**	**	**	**	**	**	**
Interactions												
I × G	**	**	NS	NS	NS	NS	NS	NS	NS	NS	NS	NS
G × K	**	**	*	*	NS	NS	NS	NS	NS	NS	NS	NS
I × K	NS	NS	NS	NS	NS	NS	NS	NS	NS	NS	NS	NS
I × G × K	NS	NS	NS	NS	NS	NS	NS	NS	NS	NS	NS	NS

^*^ = Significant at 0.05 level, ** = Significant at 0.01 level and *NS* Not significant. Means in the same column designated by the same letter are not significantly different at 5% level

The effect of genotypes was highly significant for all traits. The drought tolerance Giza178R recorded the most significant and positive values of RL, RV and RSR, while, the uppermost traits RT and RXVN assigned to L2, otherwise, RXVA belonged to L1 genotype. The kinetin treatments displayed highly significant and positively effects for all evaluated traits especially under 30 mg L^−1^ concentration. The most increased values were assigned to RV and RXVA by 14.8% and 23.9% as an average of both seasons. The interactivity between all evaluated root traits had no significant impact except the interaction between irrigation × genotypes and genotypes × kinetin regarding RL and G × K related to RV.

When analyze the intersection among genotypes × kinetin (G × K), root length (RL) and root volume (RV) had the highly significant and/or significant values with Giza 178R under 30 mg L^−1^ in both seasons (Table 5). In the same manner, the interaction between irrigation intervals and CMS lines genotypes (I × G) implied significant impacts on RL under continuous flooding (CF) with Giza178R (Table 6).

**Table 5:** Effect of interaction between genotypes and kinetin application on root length and root volume during 2020 and 2021 seasons

Genotypes	Kinetin application	R L (cm)		R V (cm3)	
				Years	
		2020	2021	2020	2021
L1	Control	18.37 h	19.35 h	31.86 h	35.36 h
	15 mg L^−1^	20.95f	20.53 g	36.48f	39.98f
	30 mg L^−1^	21.44e	22.40e	39.04de	42.59de
L2	Control	19.55 g	20.53 g	34.36 g	37.86 g
	15 mg L^−1^	22.28d	23.26d	39.65 cd	43.14 cd
	30 mg L^−1^	23.27c	24.25c	40.96bc	44.46bc
Giza178R	Control	19.83 g	20.81f	37.90ef	41.40ef
	15 mg L^−1^	23.78b	24.76b	41.98b	45.48b
	30 mg L^−1^	24.44a	25.50a	47.04a	50.51a

Means in the same column designated by the same letter are not significantly different at 5% level

**Table 6:** Effect of interaction between irrigation intervals and genotypes on root length during 2020 and 2021 seasons

Genotypes	Irrigation intervals	R L (cm)	
		Years	
		2020	2021
L1	CF	22.87e	23.85e
	I_6_	20.57 g	21.55 g
	I_9_	18.87i	19.85i
	I_12_	22.87e	18.59j
	I_15_	16.36 k	17.34 k
L2	CF	26.27b	27.25b
	I_6_	23.91d	24.89d
	I_9_	22.04f	23.02f
	I_12_	20.41 g	21.39 g
	I_15_	19.05i	20.03i
Giza178R	CF	26.96a	27.94a
	I_6_	25.06c	26.04c
	I_9_	22.76e	23.74e
	I_12_	20.86 g	21.84 g
	I_15_	19.61 h	20.59 h

Means in the same column designated by the same letter are not significantly different at 5% level

**Fig. 1:**
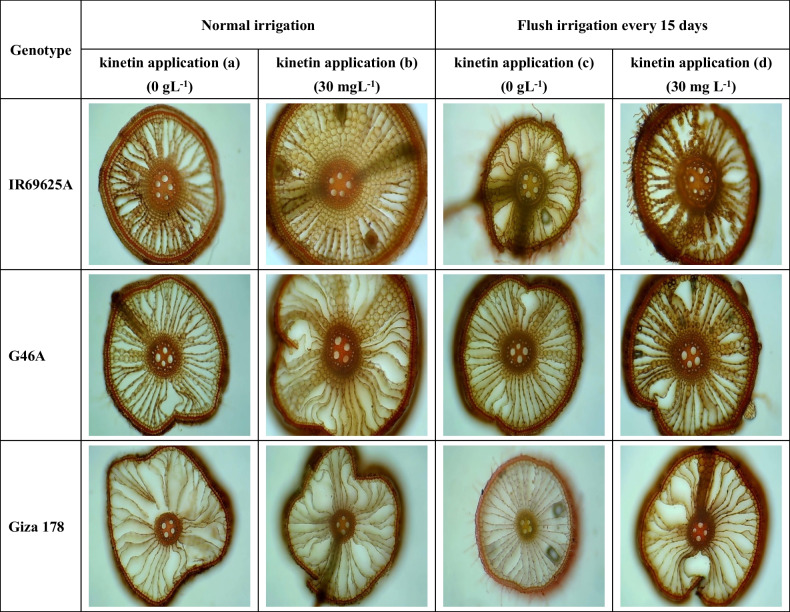
Pictorial illustration section of root xylem vessels affected by irrigation treatments coupled with or without kinetin application in rice genotypes

The anatomical characteristics in response to drought were elucidated by pictorial section. As illustrated in Fig. 1, application of the plant hormone kinetin (30gm L^−1^) increased the root cortical file number, stele diameter and xylem vessel under normal irrigation and water deficit (I_15_) (Table 6).

### Performance of floral related traits

The performance of genotypes, IR69625A (L1), G46A (L2) and Giza178R under irrigation intervening periods and kinetin employment as well as their interactions on the stigma traits (style length (SL) and total stigma length (TSL)) are presented in Table 7. The assessed traits in genotypes of interest manifested a wide variation among treatments of irrigation. CF treatment had a positive and significant effect as indicated by a decrease in floral traits values when water deficiency increased. The CMS line L1 displayed highly significant effect for SL and TSL compared to L2 and Giza 178R. Application the plant hormone kinetin at the rate of 30 mg L^−1^ positively improved both the floral traits and exhibited the highest SL and TSL values reached 19% and 4%, respectively when compared to control (non-treated plants).

**Table 7:** Effect of irrigation periods, genotypes and kinetin implementation as well as their interactions on style length and total stigma length traits the two seasons of study

Main effect and interaction	style length (SL)		Total stigma length (TSL)	
			Years	
	2020	2021	2020	2021
Irrigation intervals (I)				
CF	0.38a	0.41a	2.44a	2.45a
I_6_	0.33b	0.36b	2.23b	2.25b
I_9_	0.27c	0.30c	2.19c	2.20c
I_12_	0.24d	0.27d	2.14d	2.16d
I_15_	0.23d	0.26d	2.15d	2.16d
F-test	**	**	**	**
Genotypes (G)				
L1	0.32a	0.34a	2.53a	2.54a
L2	0.28b	0.31b	2.37b	2.39b
Giza178R	0.28b	0.31b	1.79c	1.80c
F-test	*	*	**	**
Kinetin application (K)				
Control	0.26c	0.29c	2.18c	2.21c
15 mg L^−1^	0.29b	0.32b	2.23b	2.25b
30 mg L^−1^	0.33a	0.35a	2.27a	2.30a
F-test	*	*	**	**
Interactions				
I × G	**	**	*	**
G × K	**	**	NS	NS
I × K	**	**	*	*
I × G × K	**	**	*	*

^*^ = Significant at 0.05 level, ** = Significant at 0.01 level and *NS* Not significant. Means in the same column designated by the same letter are not significantly different at 5% level

Exploring the interaction between the irrigation and genotypes (I x G) (Table 8) revealed highly significant differentiations in SL and TSL, meanwhile, I x K interplay exhibited visible changes in the measured floral traits (Table 9). Likewise, G x K interactivity exposed remarkable impact on SL but had no significant impact on TSL (Table 10). According to variance analysis, a measurable change in SL and TSL was observed by examining the reaction among I x G x K (Table 11).

**Table 8:** Effect of interaction between irrigation intervals and genotypes on style length and Total stigma length during 2020 and 2021 seasons

Lines (L)	Irrigation intervals	style length (SL)		Total stigma length (TSL)	
		Years			
		2020	2021	2020	2021
L1	CF	0.40a	0.43b	2.72a	2.74a
	I_6_	0.35b	0.38c	2.54c	2.56c
	I_9_	0.30d	0.33f	2.46d	2.48d
	I_12_	0.27e	0.30 g	2.45de	2.47de
	I_15_	0.26e	0.29 h	2.43e	2.45e
L2	CF	0.42a	0.44a	2.57b	2.59b
	I_6_	0.32c	0.36e	2.36f	2.38f
	I_9_	0.25ef	0.29 h	2.34 g	2.35 g
	I_12_	0.230 h	0.25 k	2.30 h	2.32 h
	I_15_	0.20 h	0.23 l	2.30 h	2.32 h
Giza178R	CF	0.34b	0.38c	2.01i	2.03i
	I_6_	0.32 cd	0.35e	1.78j	1.80j
	I_9_	0.27e	0.315 mg	1.74 k	1.76 k
	I_12_	0.24 fg	0.27j	1.72 l	1.73 l
	I_15_	0.23 fg	0.27j	1.71 l	1.73 l

Means in the same column designated by the same letter are not significantly different at 5% level

**Table 9:** Effect of interaction between genotypes and kinetin application on style length and total stigma length traits of both seasons

Kinetin application (K)	Irrigation Intervals	style length		Total stigma length	
		Years			
		2020	2021	2020	2021
Control	CF	0.35c	0.38c	2.38c	2.41c
	I_6_	0.27f	0.31f	2.19f	2.22f
	I_9_	0.25 g	0.28 g	2.14gh	2.15gh
	I_12_	0.22j	0.25j	2.12hi	2.14hi
	I_15_	0.20 k	0.24 k	2.10i	2.12i
15 mg L^−1^	CF	0.39b	0.42b	2.42b	2.43b
	I_6_	0.33d	0.37d	2.23e	2.25e
	I_9_	0.27f	0.30f	2.19f	2.21f
	I_12_	0.24 h	0.27 h	2.19f	2.17 g
	I_15_	0.23i	0.26i	2.15gh	2.17 g
30 mg L^−1^	CF	0.42a	0.45a	2.50a	2.52a
	I_6_	0.38b	0.42b	2.27d	2.29d
	I_9_	0.31e	0.33e	2.27d	2.29d
	I_12_	0.27f	0.30f	2.15 g	2.17 g
	I_15_	0.25 g	0.28 g	2.19f	2.21f

Means in the same column designated by the same letter are not significantly different at 5% level

**Table 10:** Effect of interaction between genotypes and kinetin application on plant style length during 2020 and 2021 seasons

Kinetin application (K)	Lines (L)	style length	
		Years	
		2020	2021
Control	L1	0.28d	0.31d
	L2	0.25e	0.28e
	Giza178R	0.25e	0.29e
15 mg L^−1^	L1	0.31b	0.34b
	L2	0.28c	0.32c
	Giza178R	0.28c	0.31 cd
30 mg L^−1^	L1	0.36a	0.39a
	L2	0.31b	0.34b
	Giza178R	0.31b	0.34b

Means in the same column designated by the same letter are not significantly different at 5% level

**Table 11:** Effect of interaction among irrigation intervals, CMS lines and kinetin application on plant floral traits during 2020 and 2021 seasons

Lines (L)	Irrigation intervals	Kinetin application	style length		Total stigma length	
					Years	
			2020	2021	2020	2021
L1	CF	Control	0.35e	0.38e	2.66b	2.68b
		15 mg L^−1^	0.40b	0.43b	2.50f	2.56e
		30 mg L^−1^	0.44a	0.47a	1.96r	1.98r
	I_6_	Control	0.39bc	0.33 h	2.50f	2.52f
		15 mg L^−1^	0.29 h	0.37ef	2.33mno	2.35mno
		30 mg L^−1^	0.43a	0.46a	1.76t	1.78t
	I_9_	Control	0.27jk	0.30jk	2.39ijk	2.41ijk
		15 mg L^−1^	0.29 h	0.33 h	2.29o	2.32o
		30 mg L^−1^	0.33f	0.36f	1.71u	1.73u
	I_12_	Control	0.24no	0.27op	2.43hi	2.45hi
		15 mg L^−1^	0.26kl	0.29kl	2.25p	2.27p
		30 mg L^−1^	0.315 mg	0.34 g	1.67u	1.69u
	I_15_	Control	0.23pq	0.26pq	2.38jkt	2.40jk
		15 mg L^−1^	0.26 lm	0.28 lm	2.25p	2.27p
		30 mg L^−1^	0.29hi	0.33hi	1.67u	1.70u
L2	CF	Control	0.39bc	0.42bc	2.69b	2.71b
		15 mg L^−1^	0.43a	0.46a	2.57cde	2.60cde
		30 mg L-1	0.43a	0.46a	1.98r	2.01r
	I_6_	Control	0.26kl	0.29kl	2.55de	2.57 cd
		15 mg L^−1^	0.33f	0.36f	2.36klm	2.38klm
		30 mg L^−1^	0.37d	0.40d	1.78st	1.81st
	I_9_	Control	0.23pq	0.26pq	2.49 fg	2.51 fg
		15 mg L^−1^	0.24no	0.27no	2.34 mn	2.36 mn
		30 mg L^−1^	0.28ij	0.31ij	1.76t	1.79t
	I_12_	Control	0.19 s	0.22 s	2.47 fg	2.48 fg
		15 mg L^−1^	0.22qr	0.25qr	2.31no	2.34no
		30 mg L^−1^	0.24op	0.27op	1.69u	1.73u
	I_15_	Control	0.17t	0.20t	2.45gh	2.47gh
		15 mg L^−1^	0.20 s	0.23 s	2.31no	2.32no
		30 mg L^−1^	0.22qr	0.26qr	1.69u	1.73u
Giza 178R	CF	Control	0.315 mg	0.34 g	2.82a	2.82a
		15 mg L^−1^	0.34ef	0.3ef	2.60c	2.62c
		30 mg L^−1^	0.38 cd	0.41 cd	2.08q	2.11q
	I_6_	Control	0.27jk	0.30jk	2.59 cd	2.61 cd
		15 mg L^−1^	0.33f	0.36f	2.40ij	2.42ij
		30 mg L^−1^	0.35e	0.38e	1.82 s	1.83 s
	I_9_	Control	0.25lmn	0.29lmn	2.50f	2.53f
		15 mg L^−1^	0.27jk	0.30jk	2.36klm	2.38klm
		30 mg L^−1^	0.30gh	0.33gh	1.78 s	1.80 s
	I_12_	Control	0.21r	0.25r	2.47 fg	2.50 g
		15 mg L^−1^	0.23pq	0.26pq	2.34lmn	2.34lmn
		30 mg L^−1^	0.26kl	0.29kl	1.76t	1.79t
	I_15_	Control	0.21r	0.25r	2.48 fg	2.515 mg
		15 mg L^−1^	0.23pq	0.26pq	2.34lmn	2.36lmn
		30 mg L^−1^	0.25mno	0.28mno	1.75t	1.76t

Means in the same column designated by the same letter are not significantly different at 5% level

In details, feasibility the interconnection between irrigation and genotypes (I x G) displayed the greatest values of SL with L2 while L1 had the maximum numbers of TSL under well-watered conditions (CF). Under severe water shortage (I_15_), the lowest numbers in SL were assigned to L2, whereas the lowest TSL exhibited in Giza178R (Table 8).

The interrelationship in between I x K regarding SL and TSL showed highly significant and/or significant increase in case of 30 mg kinetin L^−1^ implementation under well-watered treatment. A percentage reached 49.43 and 15.94 increment in SL and TSL, respectively, were detected when 30 mg kinetin L^−1^ was applied under CF comparison to un-treated plants under I_15_ (Table 9). Exploring the interaction between the genotypes and Kinetin application (G x K) revealed highly significant differences in SL. The tallest stigma was obtained by L1 when treated with 30 mg Kinetin L^−1^ with an average measured 0.375 mm (Table 10).

**Fig. 2:**
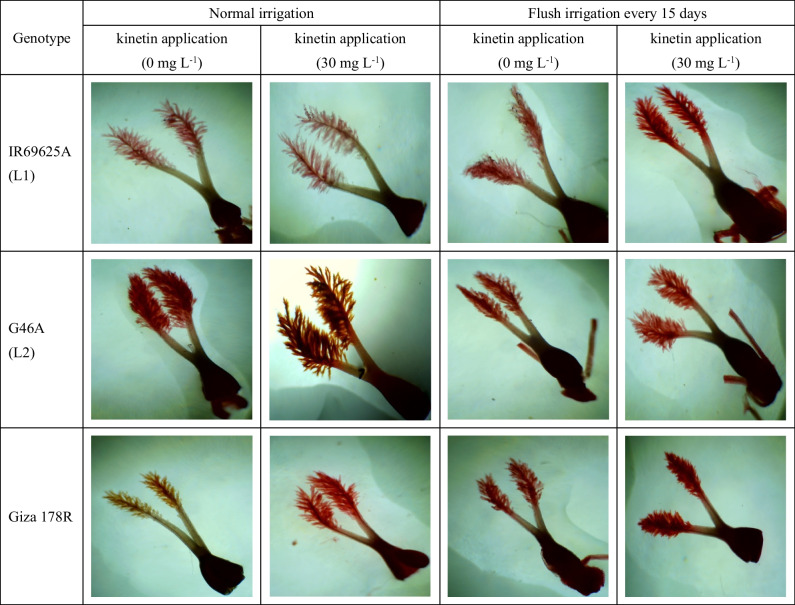
Pictorial illustration section of stigma dimensions affected by irrigation treatments with kinetin application in the three rice genotypes

In the same study, the interaction among I x L x K resulted a positive and statistically significant enhancement in SL for all irrigation times and kinetin treatments in comparison to untreated plants (Table 11 and Fig. 2). The uppermost SL values were found in L1, L2 and Giza 178R treated with 30 mg L^−1^ under all irrigation conditions. In addition, the best numbers in TSL were detected in the un-treated plants from the three genotypes under all irrigation periods.

### Yield-related traits under water deficit and kinetin application

The measured traits related to yield involve seed yield (SY), seed set (SS) and harvest index (HI) were assessed in presence of kinetin treatment, water scarcity and their interactions. The evaluated traits including SS, SY and HI showed highly significant reduction under water shortage (under I_15_) recorded 61%, 45%, and 30%, respectively, in comparison to untreated plants (Table S3). Besides, L2 X R displayed more detraction in the evaluated traits than L1 X R. Furthermore, kinetin implementation enhanced the yield-related traits (SS, SY, and HI) by 17%, 14%, and 14.5%, respectively under 30 mg kinetin L^−1^. The interactivity between I x L, L × K, I x K, and I x L × K revealed highly significant impacts in yield traits under study.

Exploring the interchange among irrigation intervals and hybrids (I x L) showed decrease in all measured traits with continue decrease in water supplementary. The reduction in seed set (SS), seed yield (SY), and harvest index (HI) reached 60.7%, 47.2%, and 30.8% for L1 X R and 61.8%, 42.7%, and 30.9% for L2 X R, respectively when I_15_ compared to CF (Table S4). When going forward to explore the interaction between the two CMS lines (Hybrids) and kinetin hormone, a valuable positive and significant increment was detected under 30 mg kinetin L^−1^. A percentages reached 16.7, 14.7 and 14.5, with L1 X R and 17.5, 14.0 and 14.4, with L2 X R, were detected in SS, SY and HI, respectively (Table S5).

In case of interrelatedness among irrigation periods and kinetin treatment (I x K), an improvement in the evaluated yield traits (SS, SY and HI) was observed under 30 mg L^−1^ of kinetin from each irrigation interval as a comparison with untreated plants (Table S6). Similarly, I x L × K (irrigation times, two CMS lines, and kinetin employment) displayed a considerable increase in yield characteristics in both hybrids under all water deficit intervals and when 30 mg L^−1^ of kinetin was spayed (Table S7).

### Correlation among traits

**Fig. 3:**
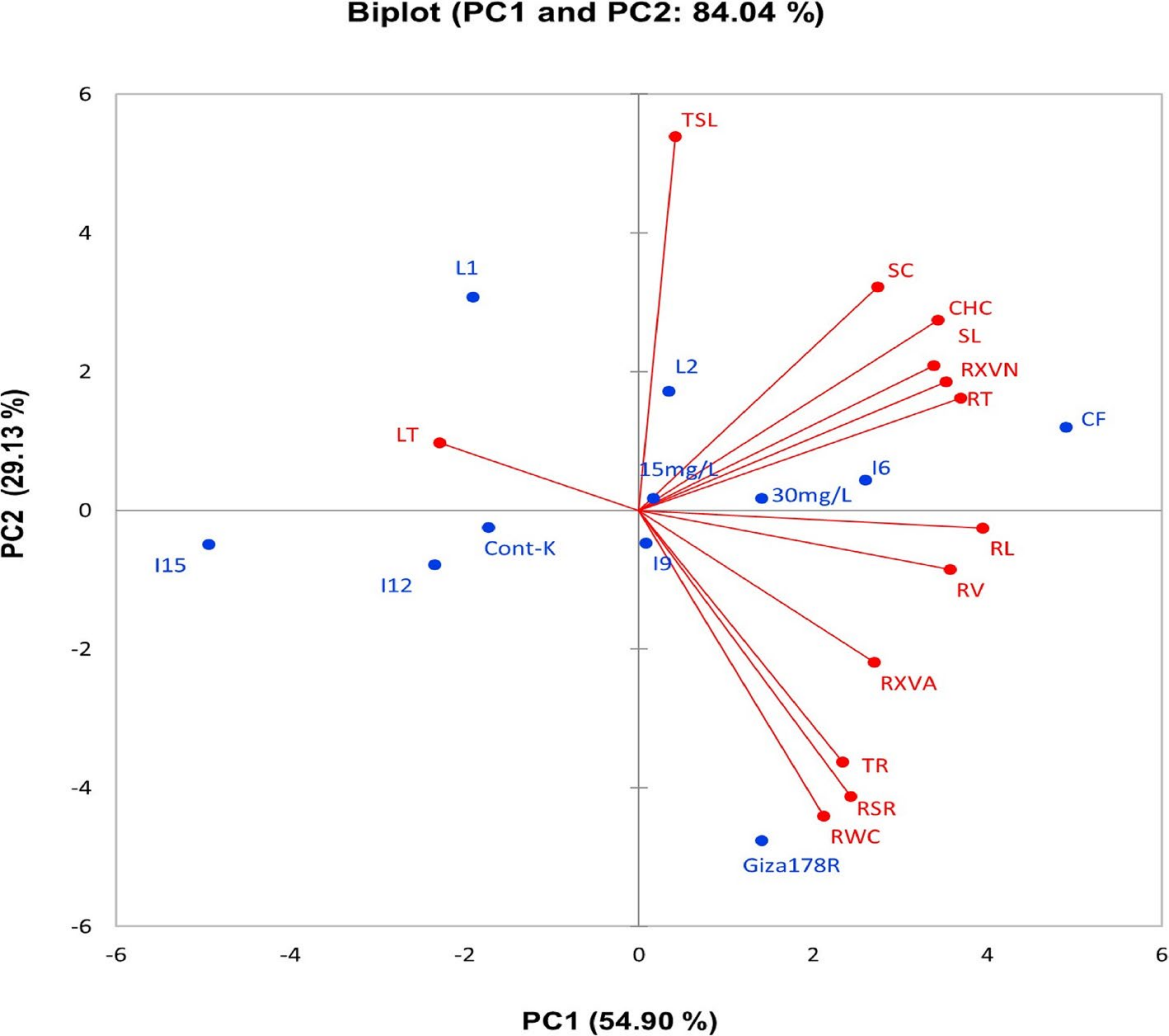
PC-biplot detecting the correlation between the evaluated traits (leaves, floral, and roots -related traits) under treatments kinetin implementation and water deficit

Estimating the association between traits under study revealed a strong correlation between the evaluated parameters as illustrated by principal component analysis. PCA1 and PCA2 calculated 84.04% (54.90% for PC1 and 29.13% for PC2) as displayed in the PC-biplot (Fig. 3). A strong Interrelationship among chlorophyl content (CHC), relative water content (RWC), stomatal conductance (SC), and transpiration rate (TR), style length (SL), root volume (RV), root length (RL), root: shoot ratio (RSR), root thickness (RT), root xylem vessel area (RXVA) and root xylem vessels number (RXVN) were detected in the positive side of PC1. Otherwise, leaf temperature (LT) exhibited a negative correlation with the other measured characteristics. Remarkably, kinetin application (15 mg L^−1^ and 30 mg L^−1^), CF, I_6_ and partially I_9_ along with L2 and Giza178R located in the positive side of PCA1 in companion with most evaluated traits.

**Fig. 4:**
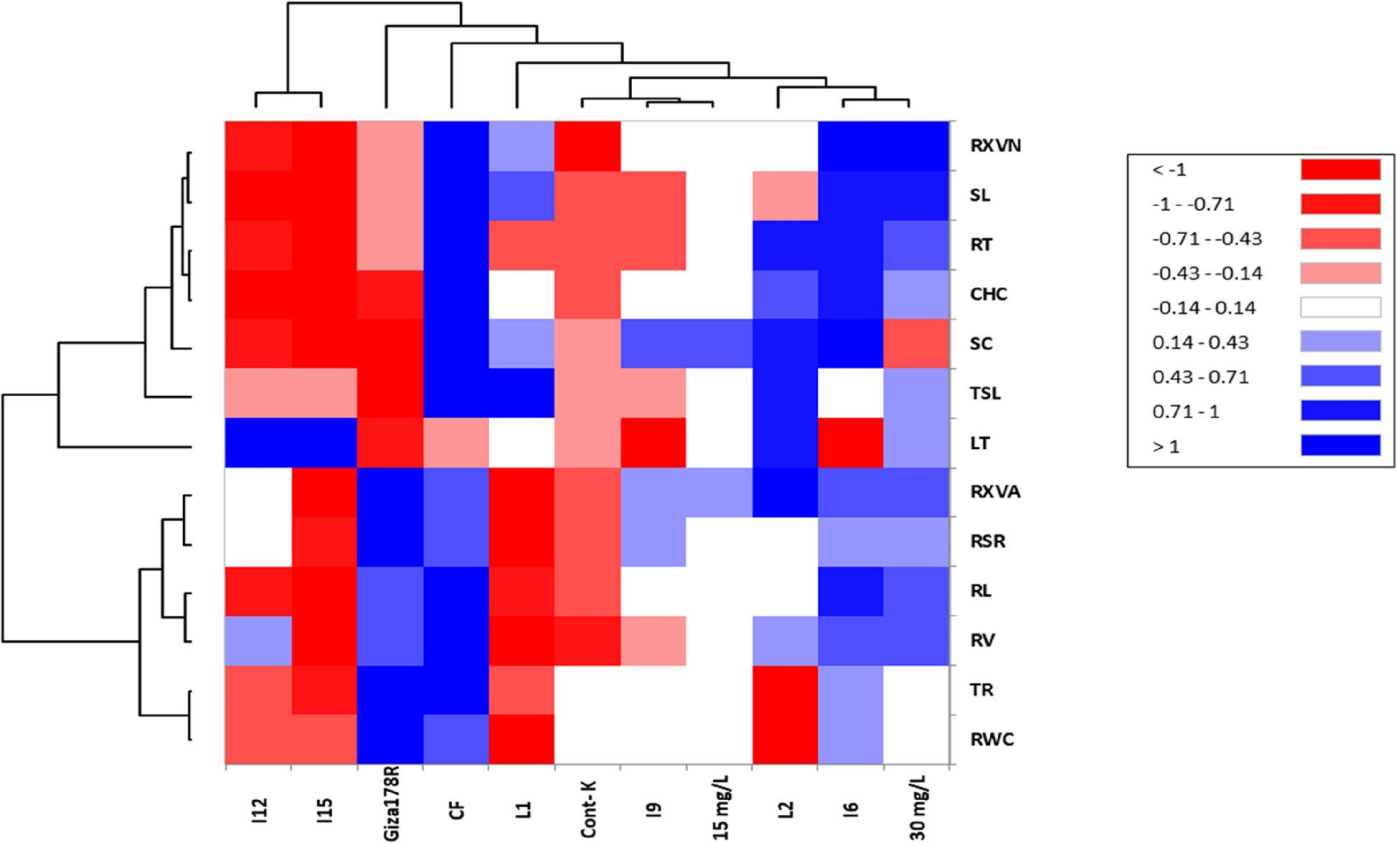
Heatmap of the correlation analysis among the implemented conditions (i.e., water intervals, CMS lines (L1, L2 and Giza178R) and kinetin application (15 and 30 mg/l) with the evaluated leaves, floral and root-related traits

To assess the interrelation among the quantified traits under water deficiency and kinetin implementation, analysis with heat map was performed (Fig. 4). Two groups of treatments were categorized (one group included I_12_ and I_15_, whereas the second group involved the rest of treatments and genotypes). Furthermore, the traits under study grouped in two categories (1^st^ group had CHC, LT, SC, SL, TSL, RT, and RXVN, while 2^nd^ group involved RWC, TR, RV, RL, RSR and RXVA). Continuous flooding (CF), I_6_ and 30 mg L^−1^ showed a positive and significant association with all evaluated traits except LT (under CF and I_6_) and SC (under 30 mg L^−1^ of kinetin). Besides, L2 and Giza178R exhibited a significant positively impact with the most characterized traits.

## Discussion

In climate change, heat and drought stresses increase in the area of crop planting that led to negative impacts on morphology, physiology, biochemical and overall yield and quality of plant. Water shortage is expected to increase and become more severe based on global temperature increases, agricultural development, industrialization and human population increment [37]. Water deficit is a clearly problem especially in arid and semi-arid regions of the world. Rice considers a sensitive plant to water deficit at all growth stages. Water scarcity in sensitive stages of rice growth will affect significantly on the plant growth, development, biochemical and physiological characteristics that will led to sustainable reduction in crop yield and quality [37–39].

Chlorophyll content (CHC), relative water content percentage (RWC%), stomatal conductance (SC), Leaf temperature (LT) and transpiration rate (TR), exhibited highly negative and significant effects under I_15_ interval. Drought has a negative impact on plant height, effective panicle number and leaf area index. Leaf area will decrease when plant suffers from water shortages through inhibiting the cell division and expansion of mesophyll tissue that drive to reduction in photosynthesis and lessening in material accumulation. Furthermore, plant height will increase by water deficit especially at the early stage of panicle differentiation whereas at mid-tillering stage, plant height will significantly reduce [40–42]. Likewise, leaf growth will minimize when plant suffers from limited water potential, subsequently, lower turgor pressure in xylem due to water deficit will guide to poor cell division and development leading finally to diminish the leaf area in rice plant [43]. Additionally, leaf anatomy and ultra-structure change under water deficiency that result reduction in number of stomata, shrinkage of leaf size, poor development of conducting system and cutinisation on leaf surface [44]. Drought prevents the development of plant organs especially the productive organs, making abnormal anther cracking, increase the flowering period and limiting the number of fertile pollen grains. On the other hand, it causes limited supply of assimilates, premature leaf senescence and shortened grouting duration during the middle and late stages of grouting [45].

Roots play a vital role in absorption of water and nutrients from soil contributing for drought tolerance [34, 46]. They prove plants and take up water and nutrients from the soil, thus, root growth strongly affects plant development and productivity. Moreover, increasing evidence indicates that root growth is deeply implicated in plant tolerance to abiotic stresses such as drought and salinity. These findings point that modifying root growth and development provides a potentially helpful approach to improve plant abiotic stress tolerance [47, 48]. In the primary stage of water deficit, plants accelerate roots growth to enhance water absorption via induction many proteins to be involved in carbon/nitrogen metabolism and root morphogenesis [49].

Under water deficiency, rice increases the length and density of root hairs as an instinctive response to face drought stress. Rice roots become more lengthen due to accumulation of abscisic acid (ABA) concentration in roots under drought stress, since rice genotype that has a deep root system, coarse roots, and capacity to produce many branches will be more tolerant to water shortages [50]. In contrary, some reports mentioned a reduction in root length in response to water deficit [51, 52]. Among rice cultivars, there are significant differences in root morphological traits (*i.e.,* root thickness, depth, and root mass) based on genetic variation in response to drought since, root architecture and growth are regulated by essential genes and metabolic processes under conditions of water-deficit [51, 53]. In peanut (*Arachis hypogaea* L.), drought stress significantly decreased root and yield characteristics (0.83–1.03 g plant^−1^ of root dry weight and 7.98–8.89 g plant^−1^ of pod dry weight), respectively [54]. Root characteristics were monitored under water deficit stress in many crops*, i.e.*, in soybean [55], wheat [56], rice [51], and common bean [57, 58].

Plant hormones such as auxin, cytokinin, abscisic acid and brassinosteroids are essential for vascular patterning and differentiation through enhancing jasmonic acid signaling. Xylem diameters and deep root growth improve water acquisition when depth water is available [51, 59–62]. Accordingly, kinetin application (30 mg L^−1^) displayed significant and/or highly positive significant impacts on all evaluated leaves traits such as CHC, RWC, SC, LT and TR when compared to untreated plants. This is supported by the findings of Malabug et al. [63] who applied kinetin externally and observed slowing down in breakdown of chlorophyll and photosynthetic proteins, which in turn delayed leaf senescence. This suggest that rice plants can be greened longer at the grain filling stage when kinetin is applied during the flowering stage, thereby, contributing to the observed increase in yield. The interaction between the irrigation periods and genotypes (I × G) showed a significant or highly significant effect for CHC, RWC%, SC and TR. Assessment of seventeen rice genotypes in presence of drought caused a noticeable reductions in chlorophyll content (CHC), relative water content (RWC), grain yield (GY), and its related-traits [64, 65]. Interaction between G x K had significant and positive effects on CHC in both seasons since kinetin hormone increased the leaf relative chlorophyll content in rice cultivar.

Performance of floral related traits such as style length (SL) and total stigma length (TSL) were affects by water deficit (Table 7). As expected, with prolonged water scarcity, the evaluated floral traits (SL and TSL) showed negative and significant decreases. The CMS line L1 produced the tallest style length and total stigma length when compared with L2 and Giza 178R. Differences among the rice genotypes in floral related traits may be attributed to the nature of the genotypes, which is mainly affected by genetic and partially by the environmental factors. Hamad et al. [66] stated that spraying some plant growth regulators (*i.e.,* gibberellic acid) in combination with different N levels can enhance stigma properties, including stigma vigor and excretion which ultimately influence the seed yield and out-crossing rate in rice. Polygenes are controlled stigma excretion in the rice genotype. Recently, part of linked QTLs were identified in relation to stigma excretion, some of these were used in QTL pyramiding to improve the hybrid rice seed production [67–69]. Tian et al., [18] reported that the excreted stigma considers the main factor affecting outcrossing rate during seed production in hybrid rice (in the female parent spikelet). Based on the spreads out of the exerted stigma at a wide angle, the area for receiving pollen will enlarge, that will led to resolve the transmitting barrier of foreign pollen, subsequently, rice genotypes can pollinate from the next day until a few days after flowering when the stigma exserted [70]. Takano-Kai et al. [71] reported a positive correlation among the stigma exertion rate and the style length in rice. Development and stigma elongation happen in the spikelets of rice pistils are basically influenced by the environment. The biological process of such traits is easy to explain, and phenotype screening is highly accurate.

Remarkably, the average of root trait values (RL, RV, RSR, RT, RXVN, and RXVA) were significantly reduced by continued exposure to water deficiency (I_15_) by 27.2%, 37.2%, 12.7%, 30.5%, 10.8%, and 32.2% if compared to CF, respectively. Kim et al. 2020 [62] reported that an extreme water deficits restrict root growth and development because of low water availability and increasing soil resistance. The primary areas of water uptake are the young root tips, despite the fact that the length and surface area of the root may influence the uptake of soil resources. Both plant productivity under drought stress and the root hydraulic conductivity are affected by the diameter of xylem vessels. When compared to plants with a larger xylem vessel diameter, those with a smaller xylem diameter typically have lower risk of cavitation and minimize hydraulic conductivity from using less water. The drought tolerance Giza178R had the highest values for RL, RV, and RSR, whereas L2 received the best values for RT and RXVN, and L1 displayed a significant increment in RXVA. These results are in consistent with those obtained by Henry et al. [72] who stated that rice genotypes with drought-tolerant and/or drought-susceptible had significantly different bleeding rates of sap from the root system. The formation lateral root increases under water deficit, that led to increase the exterior area for water absorption from contraction water columns. Additionally, there was a significant decrease in the diameter of the nodal root resulting in relatively finer roots to maintain resources. When root cross-sectional diameter increased, plant gives the priority for water retention in vascular tissue alternatively lowering radial oxygen loss in response to drought [72].

Xylem considers the main route for water and minerals transport within the plant. The xylem development relies on hormone concentration and activity of transcription factors. Abiotic stresses, such as drought and salinity represent a significant impact on xylem patterning, size and number of the water-transporting xylem vessels. Under drought stress, minimizing the risk of xylem vessel cavitation was assigned to the reduction in number or diameter of xylem vessels. Furthermore, the cell diameter of sclerenchyma get larger under water shortages since the crowded cells are not needed under drought for oxygen retention while, forming aerenchyma cells reduces because they are demanded for providing plant with oxygen in flooded soils. Genotypes with drought-resistant develop these traits to enhance water uptake along the day when effective transpiration is happen [61, 62, 72–74].

## Conclusion

Water deficit is considered a main factor in reducing crop production. Drought (irrigation every 6, 9, 12, and 15 days) is extremely injurious to rice plants and affecting the leaves, floral, and root characters. Exogenous implementation of the phytohormone kinetin (30 mg L^−1^) reduced the negative impacts of water shortage and improved most of studied traits in two CMS lines (IR69625A and G46A) during seed production in hybrid rice, respectively.

### Supplementary Information

**Additional file 1.**

## Data Availability

The dataset supporting the conclusions of this article is included within the article.

## References

[1] Shahbandeh M. Worldwide Production of Grain in 2021/22, by Type. Statista. 2022; United Kingdom. https://www.statista.com/statistics/263977/world-grain-production-by-type.

[2] Bhavsar S, Solanki T, Amin S, Jain N (2015). Assessment of genetic purity of parental lines of hybrid rice using DNA-Based markers. Online J Biol Sci.

[3] Abo-Yousef M (2018). Application for hybrid rice technology at Egypt. Ann Agric Sci Moshtohor.

[4] Virmani S, Hossain M, Bayarsaihan T. Policy Support Needs of Hybrid Rice Technology in ASia. Los Baños, Philippines: IRRi Ltd. Proc. Ser; 2006.

[5] Rout D, Jena D, Singh V, Kumar M, Arsode P, Singh P, Katara JL, Samantaray S, Verma R. Hybrid rice research: current status and prospects. In; Ansari M-R, Ed. IntechOpen: Rijeka, 2020; p. Ch. 2 ISBN 978–1–83881–032–0.

[6] Kang H, Sridhar V, Mainuddin M, Trung LD (2021). Future rice farming threatened by drought in the lower Mekong basin. Sci Rep.

[7] Korres NE, Norsworthy JK, Burgos NR, Oosterhuis DM (2017). Temperature and drought impacts on rice production: an agronomic perspective regarding short- and long-term adaptation measures. Water Resour Rural Dev.

[8] Pantuwan G, Fukai S, Cooper M, Rajatasereekul S, O’Toole JC (2002). Yield response of rice (Oryza sativa L.) genotypes to different types of drought under rainfed lowlands: Part 1. Grain yield and yield components. F Crop Res.

[9] Oladosu Y, Rafii MY, Samuel C, Fatai A, Magaji U, Kareem I, Kamarudin ZS, Muhammad I, Kolapo K (2019). Drought resistance in rice from conventional to molecular breeding: a review. Int J Mol Sci.

[10] Nie S, Mo S, Gao T, Yan B, Shen P, Kashif M, Zhang Z, Li J, Jiang C (2023). Coupling effects of nitrate reduction and sulfur oxidation in a subtropical marine mangrove ecosystem with Spartina alterniflora invasion. Sci Total Environ..

[11] Tian H, Huang N, Niu Z, Qin Y, Pei J, Wang J. Mapping winter crops in china with multi-source satellite imagery and phenology-based algorithm. Remote Sens. 2019;11(7):820. 10.3390/rs11070820.

[12] Swain P, Raman A, Singh SP, Kumar A (2017). Breeding drought tolerant rice for shallow rainfed ecosystem of eastern India. F Crop Res.

[13] Hoekstra AY, Chapagain AK, Aldaya MM, Mekonnen M. Environmental monitoring commons, hydraulic engineering commons, hydrology commons, natural resource economics commons, natural resources and conservation commons. Na. Resour Manag Policy Commons. Digital Commons Network. USA. 2011.

[14] Zhang J, Zhang S, Cheng M, Jiang H, Zhang X, Peng C, Lu X, Zhang M, Jin J (2018). Effect of drought on agronomic traits of rice and wheat: a meta-analysis. Int J Environ Res Public Health.

[15] Gewaily EE, Hamad HS, Mikhael BB, Arafat EF (2021). Performance of hybrid rice genotypes under different irrigation intervals. Menoufia J Plant Prod.

[16] Hu F, Qiu L, Xiang Y, Wei S, Sun H, Hu H, Weng X, Mao L, Zeng M (2023). Spatial network and driving factors of low-carbon patent applications in China from a public health perspective. Front Public Heal.

[17] Guo B, Wang Y, Feng Y, Liang C, Tang L, Yao X, Hu F (2022). The effects of environmental tax reform on urban air pollution: a quasi-natural experiment based on the environmental protection tax law. Front. Public Heal.

[18] Tian DC, Huang SK, Duan YG, Wang YH (2004). The relationship between flowering and pollination time and outcrossing rate of male sterile lines in hybrid rice seed production. Hybrid Rice.

[19] Ahmad HM, Wang X, Ijaz M, Mahmood-Ur-Rahman, Oranab S, Ali MA, Fiaz S. Molecular aspects of microRNAs and phytohormonal signaling in response to drought stress: a review. Curr Issues Mol Biol. 2022;44:3695–3710. 10.3390/cimb44080253 PMC9406886 36005149

[20] Akhtar SS, Mekureyaw MF, Pandey C, Roitsch T (2020). Role of cytokinins for interactions of plants with microbial pathogens and pest insects. Front. Plant Sci.

[21] Su Y, Xia S, Wang R, Xiao L (2017). Phytohormonal quantification based on biological principles. Horm Metab Signal plants.

[22] Ahmad P, Latef AAA, Hashem A, Abd Allah EF, Gucel S, Tran LSP (2016). Nitric oxide mitigates salt stress by regulating levels of osmolytes and antioxidant enzymes in chickpea. Front Plant Sci.

[23] Yadav B, Jogawat A, Gnanasekaran P, Kumari P, Lakra N, Lal SK, Pawar J, Narayan OP, Chhaya (2021). An overview of recent advancement in phytohormones-mediated stress management and drought tolerance in crop plants. Plant Gene.

[24] Werner T, Motyka V, Strnad M, Schmülling T (2001). Regulation of plant growth by cytokinin. Proc Natl Acad Sci.

[25] Novakova M, Dobrev P, Motyka V, Gaudinova A, Malbeck J, Pospisilova J, Haisel D, Storchova H, Dobra J, Mok MC (2007). Cytokinin function in drought stress response and subsequent recovery BT - biotechnology and sustainable agriculture 2006 and beyond.; Xu, Z., Li, J., Xue, Y., Yang, W., Eds..

[26] Cheng M, Cui Y, Yan X, Zhang R, Wang J, Wang X (2022). Effect of dual-modified cassava starches on intelligent packaging films containing red cabbage extracts. Food Hydrocoll.

[27] Lu L, Zhai X, Li X, Wang S, Zhang L, Wang L, Jin X, Liang L, Deng Z, Li Z (2022). Met1-specific motifs conserved in OTUB subfamily of green plants enable rice OTUB1 to hydrolyse Met1 ubiquitin chains. Nat Commun.

[28] Lazar T, Taiz L, Zeiger E (2003). Plant physiology. 3rd edn. Ann Bot.

[29] Bielach A, Hrtyan M, Tognetti VB. Plants under stress: involvement of auxin and cytokinin. Int J Mol Sci. 2017;18. 10.3390/ijms18071427. 10.3390/ijms18071427 PMC5535918 28677656

[30] Barrs HD, Weatherley PE (1962). A re-examination of the relative turgidity technique for estimating water deficits in leaves. Aust J Biol Sci.

[31] Hall AE, Lange OL, Schulze ED, Walz H. LI-1600 steady state promoter instruction manual.; October (8210–0030); LI-COR, Inc.: Lincoln, NE, USA, 1989. P16.

[32] Jagadish SVK, Muthurajan R, Oane R, Wheeler TR, Heuer S, Bennett J, Craufurd PQ (2010). Physiological and proteomic approaches to address heat tolerance during anthesis in rice (Oryza Sativa L.). J Exp Bot.

[33] Pantuwan G, Fukai Sh, Cooper M, O’Toole JC, Sarkarung S (1997). Root traits to increase drought resistance in rainfed lowland rice. Breeding strategies for rainfed lowland rice in drought-prone environments.

[34] Gaballah MM, Ghoneim AM, Ghazy MI, Mohammed HM, Sakran RM, Rehman HU, Shamsudin NAA (2021). Root traits responses to irrigation intervals in rice (Oryza Sativa). Int J Agric Biol.

[35] Stern, R.D. Statistical Procedures in Agricultural Research, By K. A. Gomez and A. A. Gomez. New York, Chichester, Etc.: Wiley (1984), 2nd Edition, Paperback, Pp. 680, Price Not Stated. Exp Agric. 1986;22:313, 10.1017/S0014479700014496.

[36] Addinsoft XLSTAT (2019). Statistical and data analysis solution.

[37] Yang Y, Yu J, Qian Q, Shang L (2022). Enhancement of Heat and drought stress tolerance in rice by genetic manipulation: a systematic review. Rice.

[38] Panda D, Mishra SS, Behera PK (2021). Drought tolerance in rice: focus on recent mechanisms and approaches. Rice Sci.

[39] Singh CM, Kumar B, Mehandi S, Chandra K (2012). Effect of drought stress in rice: a review on morphological and physiological characteristics. Trends Biosci.

[40] Alou IN, Steyn JM, Annandale JG, Van der Laan M (2018). Growth, phenological, and yield response of upland rice (Oryza sativa L. cv. Nerica 4®) to water stress during different growth stages. Agric Water Manag.

[41] Davatgar N, Neyshabouri MR, Sepaskhah ALIR, Soltani A (2009). Physiological and morphological responses of rice (Oryza sativa L) to varying water stress management strategies.

[42] Anjum SA, Ashraf U, Zohaib A, Tanveer M, Naeem M, Ali I, Tabassum T, Nazir U (2017). Growth and development responses of crop plants under drought stress: a review. Zemdirbyste.

[43] Hussain HA, Hussain S, Khaliq A, Ashraf U, Anjum SA, Men S, Wang L (2018). Chilling and drought stresses in crop plants: implications, cross talk, and potential management opportunities. Front Plant Sci.

[44] Rollins JA, Habte E, Templer SE, Colby T, Schmidt J, Von Korff M (2013). Leaf proteome alterations in the context of physiological and morphological responses to drought and heat stress in barley (Hordeum vulgare L.). J Exp Bot.

[45] Prathap V, Ali K, Singh A, Vishwakarma C, Krishnan V, Chinnusamy V, Tyagi A (2019). Starch accumulation in rice grains subjected to drought during grain filling stage. Plant Physiol Biochem.

[46] Shoaib M, Banerjee BP, Hayden M, Kant S (2022). Roots’ drought adaptive traits in crop improvement. Plants.

[47] Zhang K, Liu Y, Luo L, Zhang X, Li G, Wan Y, Liu F (2021). Root traits of peanut cultivars with different drought resistant under drought stress at flowering and pegging phase. Acta Agric Scand Sect B — Soil Plant Sci.

[48] Seo DH, Seomun S, Choi YD, Jang G (2020). Root development and stress tolerance in rice: the key to improving stress tolerance without yield penalties. Int J Mol Sci.

[49] Jaleel CA, Manivannan P, Wahid A, Farooq M, Al-Juburi HJ, Somasundaram R, Panneerselvam R (2009). Drought stress in plants: a review on morphological characteristics and pigments composition. Int J Agric Biol.

[50] Manivannan P, Jaleel CA, Sankar B, Kishorekumar A, Somasundaram R, Lakshmanan GMA, Panneerselvam R (2007). Growth, biochemical modifications and proline metabolism in Helianthus annuus L. as induced by drought stress. Colloids Surfaces B Biointerfaces..

[51] Comas LH, Becker SR, Cruz VMV, Byrne PF, Dierig DA (2013). Root traits contributing to plant productivity under drought. Front Plant Sci.

[52] Bhandari U, Gajurel A, Khadka B, Thapa I, Chand I, Bhatta D, Poudel A, Pandey M, Shrestha S, Shrestha J (2023). Morpho-physiological and biochemical response of rice (*Oryza sativa* L.)) to drought stress: a review. Heliyon.

[53] Kang J, Peng Y, Xu W (2022). Crop root responses to drought stress: molecular mechanisms, nutrient regulations, and interactions with microorganisms in the Rhizosphere. Int J Mol Sci.

[54] Junjittakarn J, Girdthai T, Jogloy S, Vorasoot N, Patanothai A (2014). Response of root characteristics and yield in peanut under terminal drought condition. Chil J Agric Res.

[55] Dayoub E, Lamichhane JR, Debaeke P, Maury P. Genotypic differences in root traits to design drought-avoiding soybean ideotypes. OCL. 2022;29:26–40. 10.1051/ocl/2022021.

[56] Bacher H, Sharaby Y, Walia H, Peleg Z. Modifying root/shoot ratios improves root water influxes in wheat under drought stress. bioRxiv 2021, 2021.08.04.455065. 10.1101/2021.08.04.455065. 10.1093/jxb/erab500 34791149

[57] Polania J, Rao IM, Cajiao C, Grajales M, Rivera M, Velasquez F, Raatz B, Beebe SE. Shoot and root traits contribute to drought resistance in recombinant inbred lines of MD 23–24 × SEA 5 of common bean. Front Plant Sci. 2017;8. 10.3389/fpls.2017.00296. 10.3389/fpls.2017.00296 PMC5334335 28316609

[58] Camilo S, Odindo AO, Kondwakwenda A, Sibiya J (2021). Root traits related with drought and phosphorus tolerance in common bean (Phaseolus vulgaris L.). Agronomy.

[59] Cornelis S, Hazak O (2022). Understanding the root xylem plasticity for designing resilient crops. Plant Cell Environ.

[60] Ramachandran P, Augstein F, Nguyen V, Carlsbecker A (2020). Coping with water limitation: hormones that modify plant root xylem development. Front Plant Sci.

[61] Prince SJ, Murphy M, Mutava RN, Durnell LA, Valliyodan B, Shannon JG, Nguyen HT (2017). Root xylem plasticity to improve water use and yield in water-stressed soybean. J Exp Bot.

[62] Kim Y, Chung YS, Lee E, Tripathi P, Heo S, Kim K-H (2020). Root response to drought stress in rice (Oryza sativa L.). Int J Mol Sci.

[63] Nueve L, Sta. Cruz P, Banayo NPM, Aguilar E, Hernandez J. Improving the Grain Filling and Yield of Indica Rice through Kinetin (N-6-furfuryl adenine) Application at Flowering Stage. Crop Prot Newsl. 2010;35:22–35.

[64] Kadam NN, Tamilselvan A, Lawas LMF, Quinones C, Bahuguna RN, Thomson MJ, Dingkuhn M, Muthurajan R, Struik PC, Yin X (2017). Genetic control of plasticity in root morphology and anatomy of rice in response to water deficit. Plant Physiol.

[65] Villa JE, Henry A, Xie F, Serraj R (2012). Hybrid rice performance in environments of increasing drought severity. F Crop Res.

[66] Hamad HSH, Ghoneim A, Gomaa M (2021). Effect of n levels and some plant growth regulators on hybrid rice (Oryza Sativa L.) seed production. Appl Ecol Environ Res.

[67] Qi B, Wu C (2022). Potential roles of stigma exsertion on spikelet fertility in rice (Oryza sativa L.) under heat stress. Front. Plant Sci.

[68] Hamad HS, Bleih EM, Gewaily EE, Alharbi K, Rehan M (2023). The potential effects of kinetin implementation on hybrid rice seed production under water deficit. Sustainability.

[69] Barik SR, Pandit E, Mohanty SP, Nayak DK, Pradhan SK (2020). Genetic mapping of physiological traits associated with terminal stage drought tolerance in rice. BMC Genet.

[70] Jiang JH, Zhang WH, Dang XJ, Rong H, Ye Q, Hu CM, Zhang Y, He Q, Wang DZ (2021). Genetic analysis of stigma traits with genic male sterile line by mixture model of major gene plus polygene in rice (Oryza sativa L.). Acta Agron Sin.

[71] Takano-Kai N, Doi K, Yoshimura A (2011). GS3 participates in stigma exsertion as well as seed length in rice. Breed Sci.

[72] Henry A, Cal AJ, Batoto TC, Torres RO, Serraj R (2012). Root attributes affecting water uptake of rice (Oryza sativa) under drought. J Exp Bot.

[73] Gowayed Salah MH, Diaa AEM, MetwaliEhab MR, El-Malky Mohamed M (2020). Combining ability and heterosis studies for some economic traits in rice (Oryza sativa L.). Res J Biotechnol.

[74] ElShamey EAZ, Sakran RM, ElSayed MAA, Aloufi S, Alharthi B, Alqurashi M, Mansour E, Abd El-Moneim D (2022). Heterosis and combining ability for floral and yield characters in rice using cytoplasmic male sterility system. Saudi J Biological Sci.

