# Supplementary material for: Impacts of kinetin implementation on leaves, floral and root-related traits during seed production in hybrid rice under water deficiency
Source: BMC Plant Biol. 2023 Aug 22;23:398. doi: 10.1186/s12870-023-04405-6 (PMC10463769; doi:10.1186/s12870-023-04405-6)
Supplement: Supplementary file 1 — Additional file 1. [file 12870_2023_4405_MOESM1_ESM.docx]

Table S1: Monthly average minimum temperature, maximum temperature and relative humidity at the experimental site in the two summer seasons of 2020 and 2021.

| Month | Minimum temperature (°C) | | Maximum temperature (°C) | | Relative humidity (%) | |
| --- | --- | --- | --- | --- | --- | --- |
|  | 2020 | 2021 | 2020 | 2021 | 2020 | 2021 |
| April | 18.9 | 18.62 | 26 | 30.03 | 59.55 | 58.70 |
| May | 23.8 | 22.81 | 31.9 | 30.4 | 50.65 | 55.40 |
| June | 25.2 | 26.3 | 31.1 | 33.6 | 57.30 | 58.15 |
| July | 27.3 | 26.1 | 33.7 | 33.7 | 67.65 | 69.75 |
| August | 28.2 | 26 | 34.6 | 33.6 | 67.45 | 70.30 |
| Septemper | 27.1 | 24.3 | 34 | 32.6 | 67.20 | 67.45 |
| October | 24.6 | 21.5 | 34.6 | 29.5 | 65.95 | 68.85 |

Table S2: Some chemical and physical properties of experimental soil in Sakha

| **Properities** | **Sakha** | |
| --- | --- | --- |
|  | **2020** | **2021** |
| pH | 7.9 | 8.1 |
| ECe dS.m^-1^ | 2.3 | 2.5 |
| O.M. % | 1.15 | 1.2 |
| Available N, mg kg^-1^ | 32 | 31 |
| Available P, mg kg^-1^ | 13 | 12 |
| Available K, mg kg^-1^ | 420 | 410 |
| Soluble cations meq. L^-1^ | - | - |
| Ca^++^ | 5.0 | 5.2 |
| Mg^++^ | 3.0 | 4.0 |
| K^+^ | 0.3 | 0.40 |
| Na^+^ | 14.7 | 15.5 |
| Soluble anions meq. L^-1^ | - | - |
| CO3-- | - | - |
| HCO3 | 3.0 | 4.0 |
| CL- | 16.0 | 16.2 |
| SO4-- | 4.0 | 5.2 |
| Available micronutrients ppm | - | - |
| Fe^++^ | 5.7 | 5.2 |
| Zn^++^ | 1.12 | 1.21 |
| Mn^++^ | 4.7 | 4.3 |

Table S3. Effect of irrigation periods, two hybrids and kinetin application as well as their interactions on seed yield (SY), seed set (SS) and harvest index (HI) during 2020 and 2021 seasons.

| Main effect and interaction | **SS (%)** | | **SY (t ha^-1^)** | | **HI (%)** | |
| --- | --- | --- | --- | --- | --- | --- |
|  | 2020 | 2021 | 2020 | 2021 | 2020 | 2021 |
| Irrigation intervals (I) |  |  |  |  |  |  |
| CF | 31.96a | 37.26a | 1.728a | 1.839a | 18.98a | 19.94a |
| I_6_ | 25.89b | 28.71b | 1.395b | 1.503b | 17.38b | 18.67b |
| I_9_ | 21.70c | 24.44c | 1.289c | 1.371c | 16.25c | 17.42c |
| I_12_ | 19.37d | 22.02d | 1.194d | 1.264d | 15.00d | 16.15d |
| I_15_ | 12.16e | 14.68e | 0.939e | 1.021e | 12.85e | 14.06e |
| F-test | ** | ** | ** | ** | ** | ** |
| CMS lines (L) |  |  |  |  |  |  |
| L1 X R | 23.05a | 26.68a | 1.354a | 1.439a | 16.45a | 17.61a |
| L2 X R | 21.38b | 24.16b | 1.264b | 1.361b | 15.64b | 16.89b |
| F-test | * | ** | ** | ** | ** | ** |
| Kinetin application (K) |  |  |  |  |  |  |
| Control | 19.82c | 23.52c | 1.222c | 1.298c | 14.81c | 15.74c |
| 15mg L^-1^ | 22.10b | 25.25b | 1.281b | 1.384b | 16.28b | 17.47b |
| 30mg L^-1^ | 24.73a | 27.51a | 1.425a | 1.517a | 17.18a | 18.53a |
| F-test | ** | ** | ** | ** | ** | ** |
| Interactions |  |  |  |  |  |  |
| I x L | ** | ** | ** | ** | * | ** |
| L× K | ** | ** | ** | ** | ** | ** |
| I x K | ** | ** | ** | ** | ** | ** |
| I x L × K | ** | * | ** | ** | ** | ** |

*, ** Significant and highly significant at the 1% level of probability. NS not significant at the 1% level of probability.

Table S4. Effect of interaction between CMS lines and irrigation periods on seed yield (SY), seed set (SS) and harvest index (HI) during 2020 and 2021 seasons.

| CMS Lines | Irrigation  intervals | SS (%) | | SY (t ha^-1^) | | HI (%) | |
| --- | --- | --- | --- | --- | --- | --- | --- |
|  |  | 2020 | 2021 | 2020 | 2021 | 2020 | 2021 |
| L1 X R | CF | 31.92a | 39.40a | 1.850a | 1.935a | 19.23a | 20.44a |
|  | I_6_ | 27.29a | 30.12c | 1.424c | 1.537c | 17.88c | 19.08c |
|  | I_9_ | 22.71d | 25.46f | 1.312e | 1.391e | 16.82d2 | 17.78e |
|  | I_12_ | 20.55e | 23.15f | 1.226g | 1.290g | 15.57e | 16.48g |
|  | I_15_ | 12.79g | 15.27h | 0.958i | 1.041i | 13.215mg | 14.26i |
| L2 X R | CF | 32.00a | 35.12b | 1.606b | 1.744b | 18.73b | 19.44b |
|  | I_6_ | 24.50c | 27.31d | 1.365d | 1.468d | 16.88d | 18.26d |
|  | I_9_ | 20.69e | 23.42f | 1.267f | 1.351f | 15.68e | 17.06f |
|  | I_12_ | 18.19f | 20.87g | 1.162h | 1.238h | 14.43f | 15.81h |
|  | I_15_ | 11.53h | 14.09i | 0.921j | 1.000j | 12.49h | 13.87j |

Table S5. Effect of interaction between two CMS lines and kinetin application on seed yield (SY), seed set (SS) and harvest index (HI) during 2020 and 2021 seasons.

| CMS lines | Kinetin application | SS (%) | | SY (t ha^-1^) | | HI (%) | |
| --- | --- | --- | --- | --- | --- | --- | --- |
|  |  | 2020 | 2021 | 2020 | 2021 | 2020 | 2021 |
| L1 X R | Control | 20.29e | 25.00c | 1.262d | 1.331e | 15.21d | 16.02e |
|  | 15mg L^-1^ | 23.06c | 26.49b | 1.320c | 1.424c | 16.85b | 17.85c |
|  | 30mg L^-1^ | 25.80a | 28.55a | 1.479a | 1.562a | 17.59a | 18.95a |
| L2 X R | Control | 19.34f | 22.03e | 1.180f | 1.265f | 14.41e | 15.46f |
|  | 15mg L^-1^ | 21.14d | 24.00d | 1.242e | 1.3455d | 15.72c | 17.10d |
|  | 30mg L^-1^ | 23.66b | 26.46b | 1.372b | 1.472b | 16.79b | 18.11b |

Table S6. Effect of interaction between Irrigation intervals and kinetin application on seed yield (SY), seed set (SS) and harvest index (HI) during 2020 and 2021 seasons.

| Irrigation  Intervals | Kinetin  application | SS (%) | | GY (t ha^-1^) | | HI (%) | |
| --- | --- | --- | --- | --- | --- | --- | --- |
|  |  | 2020 | 2021 | 2020 | 2021 | 2020 | 2021 |
| CF | Control | 28.8d | 36.65c | 1.687b | 1.753c | 18.36d | 18.44d |
|  | 15mg L^-1^ | 32.0b | 37.17b | 1.694b | 1.861b | 18.99b | 20.23b |
|  | 30mg L^-1^ | 34.96a | 37.95a | 1.803a | 1.904a | 19.58a | 21.15a |
| I_6_ | Control | 22.0g | 24.80g | 1.233h | 1.325g | 15.57g | 16.64f |
|  | 15mg L^-1^ | 25.09e | 27.89e | 1.329e | 1.423e | 17.90d | 19.23c |
|  | 30mg L^-1^ | 30.54c | 33.44d | 1.623c | 1.762c | 18.66c | 20.15b |
| I_9_ | Control | 19.60i | 22.30i | 1.189i | 1.262h | 14.46i | 15.55h |
|  | 15mg L^-1^ | 21.7gh | 24.530mgh | 1.2815mg | 1.380f | 16.50f | 17.52e |
|  | 30mg L^-1^ | 23.72f | 26.50f | 1.398d | 1.472d | 17.79e | 19.18c |
| I_12_ | Control | 17.12j | 19.78j | 1.110k | 1.182j | 13.49j | 14.65i |
|  | 15mg L^-1^ | 19.45i | 21.98i | 1.167i | 1.237i | 15.16h | 16.30g |
|  | 30mg L^-1^ | 21.54h | 24.28h | 1.305f | 1.374f | 16.35f | 17.50e |
| I_15_ | Control | 11.4m | 14.04m | 0.888n | 0.96m | 12.16l | 13.42k |
|  | 15mg L^-1^ | 12.09l | 14.65l | 0.93m | 1.023l | 12.86k | 14.10j |
|  | 30mg L^-1^ | 12.9k | 15.35k | 0.966l | 1.072k | 13.52j | 14.68j |

Table S7. Effect of interaction among irrigation intervals, two CMS lines and kinetin application on seed yield (SY), seed set (SS) and harvest index (HI) traits during 2020 and 2021 seasons.

| Irrigation intervals | CMS lines | Kinetin application | SS (%) | | SY (t ha^-1^) | | HI (%) | |
| --- | --- | --- | --- | --- | --- | --- | --- | --- |
|  |  |  | 2020 | 2021 | 2020 | 2021 | 2020 | 2021 |
| CF | L1 X R | Control | 26.44f | 39.26ab | 1.783c | 1.851c | 18.36d | 18.81e |
|  |  | 15mg L^-1^ | 32.53bc | 39.11b | 1.815b | 1.953b | 19.5ab | 20.64b |
|  |  | 30mg L^-1^ | 36.81a | 39.82a | 1.952a | 1.998a | 19.77a | 21.85a |
|  | L2 X R | Control | 26.44f | 34.04e | 1.560f | 1.655g | 18.36d | 18.08f |
|  |  | 15mg L^-1^ | 31.66cd | 25.25d | 1.604e | 1.766e | 18.43d | 19.81d |
|  |  | 30mg L^-1^ | 33.11b | 36.08c | 1.654d | 1.810d | 19.39b | 20.44c |
| I_6_ | L1 × R | Control | 23.76g | 26.53i | 1.256l | 1.347k | 16.15g | 16.90i |
|  |  | 15mg L^-1^ | 26.66f | 29.49g | 1.343i | 1.445i | 18.41d | 19.67d |
|  |  | 30mg L^-1^ | 31.44d | 34.34e | 1.674d | 1.819d | 19.08c | 20.67b |
|  | L2 X R | Control | 20.35j | 23.08k | 1.211m | 1.302l | 15.00i | 16.38j |
|  |  | 15mg L^-1^ | 23.51hi | 26.29i | 1.314jk | 1.400j | 17.40e | 18.78e |
|  |  | 30mg L^-1^ | 29.63e | 32.54f | 1.572f | 1.704f | 18.24d | 19.62d |
| I_9_ | L1 X R | Control | 20.86j | 23.58k | 1.201m | 1.278m | 15.07h | 15.87k |
|  |  | 15mg L^-1^ | 22.67i | 25.42j | 1.307k | 1.398j | 17.34e | 17.830mg |
|  |  | 30mg L^-1^ | 24.59g | 27.38h | 1.426g | 1.498h | 18.25d | 19.62d |
|  | L2 X R | Control | 18.34k | 21.02l | 1.176n | 1.246n | 13.28l | 15.24l |
|  |  | 15mg L^-1^ | 20.86j | 23.62k | 1.255l | 1.362k | 15.85g | 17.23h |
|  |  | 30mg L^-1^ | 22.85hi | 25.62j | 1.371h | 1.446i | 17.34e | 18.72e |
| I_12_ | L1 X R | Control | 18.41k | 21.08l | 1.139o | 1.197o | 13.94k | 14.89m |
|  |  | 15mg L^-1^ | 20.64j | 23.03k | 1.205m | 1.276m | 15.85g | 16.74i |
|  |  | 30mg L^-1^ | 22.61i | 25.36j | 1.334ij | 1.396j | 16.91f | 17.82i |
|  | L2 X R | Control | 15.84l | 20.95l | 1.080p | 1.166p | 13.03l | 14.42op |
|  |  | 15mg L^-1^ | 18.26k | 23.02k | 1.129o | 1.197o | 13.94k | 15.86k |
|  |  | 30mg L^-1^ | 20.46j | 23.19k | 1.277l | 1.353k | 15.79h | 17.17h |
| I_15_ | L1 X R | Control | 12.00no | 14.56p | 0.902s | 0.981t | 12.53m | 13.65q |
|  |  | 15mg L^-1^ | 12.81mn | 15.38no | 0.962r | 1.045r | 13.28l | 14.37p |
|  |  | 30mg L^-1^ | 13.55m | 15.85n | 1.010q | 1.097q | 13.82k | 14.76mn |
|  | L2 X R | Control | 10.96p | 13.51q | 0.875t | 0.955u | 11.81n | 13.18r |
|  |  | 15mg L^-1^ | 11.36op | 13.92q | 0.905s | 1.001s | 12.45m | 13.83q |
|  |  | 30mg L^-1^ | 12.28no | 14.86op | 0.983r | 1.046r | 13.22l | 14.59no |
